# Paraneoplastic Encephalopathy in a Patient With Metastatic Lung Cancer: A Case Study

**Published:** 2018-03-01

**Authors:** Scott M. Rowley

**Affiliations:** The Ohio State University Comprehensive Cancer Center – Arthur G. James Cancer Hospital and Richard J. Solove Research Institute

## Abstract

**CASE STUDY**

RS, a 36-year-old female, presented to the emergency department (ED) of a large academic medical center upon the advice of her primary care provider because of 3 weeks of progressive mental status changes, weakness, and decreased oral intake. According to her husband, RS was diagnosed with stage IIIA large cell lung cancer 8 months earlier and was treated with concurrent chemotherapy (carboplatin, pemetrexed, and bevacizumab) and radiation therapy that was completed 4 months prior to admission. No other specific information about her treatment or outside health records was available.

According to her husband, RS had been in her usual state of health until approximately 3 weeks prior, when she began having significant mental status changes. She first exhibited some difficulty finding words and later was noted to be putting food in a coffee maker. This spontaneously resolved after approximately 1 week; however, she rapidly developed slurred speech and began to make nonsensical statements. These manifestations also slowly improved but were followed by worsening speech deficit, difficulty walking, and impaired balance. During one of these episodes, she had an occurrence of incontinence. Her husband also noted an incident where her "eyes were beating back and forth and the left side of her face was twitching." RS also had periods (according to her husband) where she "did not seem to be interacting with her environment." These progressively worsened during the last week, and she completely stopped walking and talking 2 days prior to coming to the ED.

According to her husband, RS had rheumatoid arthritis and no surgical history. Her family history was unknown except that RS’s mother had "seizures." RS had reportedly not used tobacco, alcohol, or drugs, and she was sexually active with her husband. Home medications included transdermal fentanyl 12 μg/hr patch changed every 72 hours; oxycodone-acetaminophen tablets 5-325 mg, two every 4 hours as needed for pain; prednisone 10 mg, one tablet daily; and megestrol 40 mg/mL suspension, 20 mL once daily for appetite stimulation.

RS was admitted to an inpatient medical oncology service and evaluated by the oncology advanced practitioner (AP) on her second inpatient day. Upon exam, RS was nonverbal except for moaning in response to painful stimuli and to her sister’s voice. Her vital signs were normal. She appeared ill but well-nourished, and she was mildly diaphoretic. Neurologic examination revealed that her pupils were slightly sluggish but equal, round, and reactive to light. Extraocular muscle movements were intact, but she did not move her eyes in response to commands. She tracked the AP and family members around the room with her eyes. Cranial nerve examination was intact with the exception of cranial nerves IX, X, and XI, which were difficult to examine given her inability to cooperate and open her mouth. Motor examination revealed increased tone throughout and intermittent, inconsistent resistance to passive movement. She was seen to move all four extremities spontaneously although not in response to commands. Deep tendon reflexes were intact and equal in all extremities.

Examination of other body systems was as follows: there was dry, peeling skin on her lips, but her mucous membranes were moist and free of erythema or lesions. Her lungs were clear to auscultation bilaterally. Her heart rate and rhythm were regular, there were no murmurs, rubs, or gallops, and distal pulses were intact. Her abdomen was nondistended with normally active bowel sounds in all four quadrants. Her abdomen was soft, nontender to palpation, and without palpable masses. There was no peripheral discoloration, temperature changes, or edema, and examination of her skin was benign.

**Workup**

On admission to the emergency department, serum laboratory studies were unrevealing for any potential causes of encephalopathy. Kidney and liver function were normal, making diagnoses of uremic and hepatic encephalopathies less likely. Cultures of the urine and blood were negative. Samples of cerebrospinal fluid (CSF) were obtained via lumbar puncture and were unrevealing for any abnormalities.

Computed tomography (CT) of the head without contrast was negative for any acute intracranial process. Ultrasound of the right upper quadrant revealed a single, nonspecific, hypoechoic hepatic lesion. Computed tomography scans of the chest, abdomen, and pelvis demonstrated the primary malignancy in the upper lobe of the left lung, as well as possible metastatic disease within the left lung, right lung, and liver, and widespread osseous metastatic disease. Magnetic resonance imaging (MRI) of the brain performed 1 day after admission demonstrated numerous scattered punctate foci of enhancement throughout the supratentorial and infratentorial brain parenchyma, measuring at most 3 to 4 millimeters in diameter. There was no significant mass effect or midline shift. A paraneoplastic panel was sent to an outside laboratory and returned positive for antivoltage-gated potassium channel (VGKC) autoantibodies.

**Differential Diagnosis**

Clinically, RS was exhibiting signs of encephalopathy, a broad term that indicates general brain dysfunction, the hallmark of which is altered mental status. Diagnosing encephalopathy is challenging, as many differential diagnoses must be considered. The clinician must consider metabolic derangements, toxic and infectious etiologies, psychiatric disorders, and less commonly, prion disorders and progressive dementia. Cultures of RS’s blood and urine as well as other specialized endocrine tests were negative, decreasing the likelihood of a metabolic or infectious cause for her presentation. The abnormalities on her brain MRI were reviewed by a neuro-oncology team, who felt that the faint, nondescript nature of the visualized lesions was not suspicious for metastatic disease. Sequelae of seizures was also considered by neuro-oncology but dismissed given a grossly normal prolonged electroencephalogram.

Some encephalopathies are caused by autoimmune or inflammatory mechanisms, which are confirmed by the presence of autoantibody markers and/or clear response to immunomodulatory treatment (Vernino, Geschwind, & Boeve, 2007). These types of encephalopathies have been seen in patients with cancer and have thus been termed paraneoplastic. The presence of anti-VGKC antibodies on RS’s paraneoplastic panel directed the inpatient medical oncology team toward a paraneoplastic neurologic disorder (PND) as the most likely diagnosis.

Paraneoplastic syndromes are a group of disorders that are associated with malignant diseases (cancers) but are not directly related to the physical effects of the primary or metastatic tumors (Kanaji et al., 2014). The term "paraneoplastic" was coined in the 1940s; however, paraneoplastic conditions have remained poorly understood until recently. Current understanding of paraneoplastic syndromes attributes the disease states either to tumor secretion of functional peptides and hormones, or to immune cross-reactivity between tumor and normal host tissues (Pelosof & Gerber, 2010). The secretion of functional peptides and hormones by tumor tissue is responsible for endocrine paraneoplastic syndromes such as the syndrome of inappropriate antidiuretic hormone secretion, humoral hypercalcemia of malignancy, Cushing syndrome, and carcinoid syndrome.

Paraneoplastic neurologic disorder, in addition to various dermatologic, rheumatologic, and hematologic paraneoplastic syndromes, is caused by immune cross-reactivity between tumor and normal host tissues. The classification of PND is long and includes many rare neurologic conditions. Encephalomyelitis, limbic encephalitis, subacute cerebellar degeneration, opsoclonus-myoclonus syndrome, Lambert-Eaton myasthenic syndrome (LEMS), and acquired neuromyotonia are some of the more common presentations (Kanaji et al., 2014).

Few studies address the incidence and prevalence of PND in the overall population of patients with cancer. One study found that 4% of women with breast cancer, 16% of men with lung cancer, and 6.6% of patients with all cancers had evidence of PND compared with 1% to 2% of age-matched controls (Croft & Wilkinson, 1965). Data described by Rees (2004) suggest that PND usually presents later in life, with a median age of onset of 66 years. Conflicting data exist regarding the sex ratio of patients with PND, demonstrating both female and male predominance (Rees, 2004). This seems to vary based on geographic location; however, no identifiable pattern has been described.

Although the presentation of PND is possible in the setting of any malignancy, it is most commonly seen in association with cancers that express neuroendocrine proteins, affect organs with immunoregulatory properties, or contain neuronal tissue. Interestingly, cancer is not observed in about 20% of cases of PND, probably reflecting successful control of tumor growth and metastasis by the host immune system (Rees, 2004). One of the most common and notorious PND-associated malignancies is lung cancer (Dalmau & Rosenfeld, 2008). The frequency of PND in lung cancer patients may reach 30%, and the presence of well-defined onconeural antibodies has been observed in 20% of these patients (Zaborowski & Michalak, 2013; Table 1).

**Table 1:**
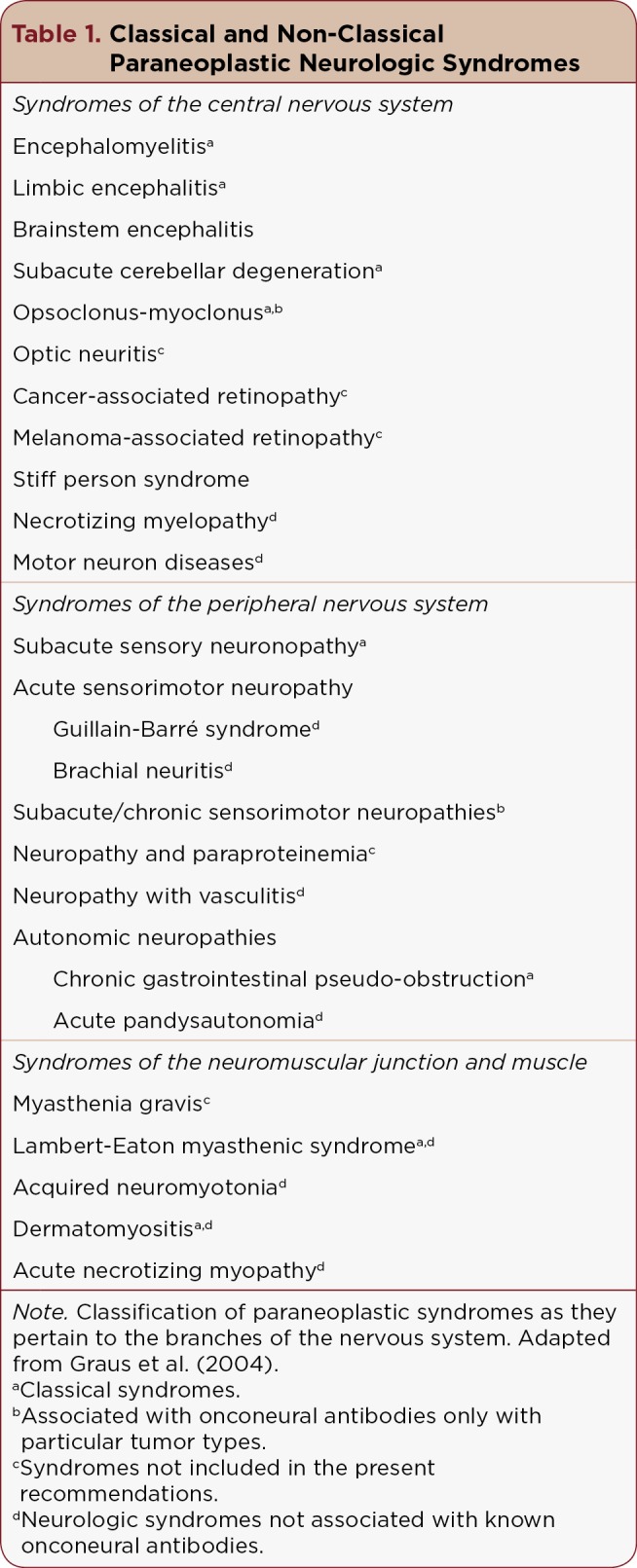
Classical and Non-Classical Paraneoplastic Neurologic Syndromes

## PATHOGENESIS

The clinical features of PND can be attributed to the presence of autoantibodies that are produced in response to a primary malignancy. These antibodies are termed "onconeural antibodies." Onconeural antibodies and associated onconeural T lymphocytes inadvertently attack both tumor cells and components of the nervous system (Kanaji et al., 2014). These antibodies react with both intracellular and extracellular antigens, including nuclear proteins and membrane channels. They are thought to arise as a result of the aberrant expression of antigens common to tumors and neurons (Rees, 2004).

Known onconeural antibodies have been reported in fewer than 50% of patients with PND; therefore, the absence of known antibodies does not rule out the presence of PND. Standard investigations such as blood tests, CT and MRI scanning, and CSF analysis are rarely useful in confirming a diagnosis of PND (Rees, 2004). The diagnosis often rests either on the demonstration of an underlying malignancy (if not already known) or on the presence of circulating onconeural antibodies, often demonstrated using a battery of serum tests commonly known as a paraneoplastic panel.

**Antivoltage-Gated PotassiumChannel Antibodies**

Many autoantibodies have been identified in patients with paraneoplastic syndromes. The paraneoplastic panel performed on the serum of our patient, RS, was positive for anti-VGKC antibodies. Voltage-gated potassium channels are highly relevant to neuronal and muscular physiology given their vital contribution to the cellular action potential. They are specifically implicated in initiating paraneoplastic autoimmunity. Serum antibodies reactive with VGKC were initially reported as markers of acquired neuromyotonia and are also found in Morvan syndrome, a rare autoimmune condition consisting of irregular contractions of the long muscles, cramping, weakness, pruritus, hyperhidrosis, severe insomnia, and delirium (Tan, Lennon, Klein, Boeve, & Pittock, 2008). While RS’s signs and symptoms do not meet all criteria for Morvan syndrome, it is reasonable to suspect that the presence of anti-VGKC autoantibodies is implicated in her clinical presentation.

## DIAGNOSIS AND MANAGEMENT

Unfortunately, patients with PND are often severely disabled at the time of diagnosis, and no attempt at treatment is effective. Immunomodulatory therapies such as corticosteroids, azathioprine, cyclophosphamide, and plasma exchange are often tried but, with the exception of the treatment of LEMS, have generally been unsuccessful (Rees, 2004). Rituximab and intravenous immunoglobulins have also been used; however, the impact of these therapies remains unclear because the number of patients treated is low, patients also receive antineoplastic therapy, and randomized prospective studies are lacking (Graus & Dalmau, 2013). Diagnosis and treatment of the underlying malignancy remains the mainstay of treatment for PND (Table 2).

**Table 2:**
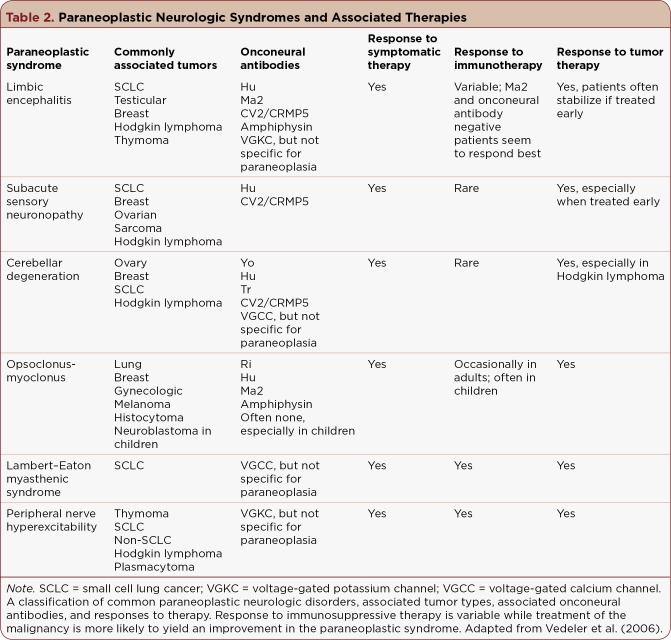
Paraneoplastic Neurologic Syndromes and Associated Therapies

## CONCLUSION

In the case of RS, a diagnosis of paraneoplastic encephalopathy was made based on her clinical presentation and the presence of anti-VGKC antibodies on her paraneoplastic panel. The medical oncology team consulted the neurology and neuro-oncology services who first recommended 5 days of high-dose IV methylprednisolone sodium succinate. When this failed to elicit a significant response, plasma exchange was pursued. Five total treatments were planned. RS received 3 treatments with no improvement. A percutaneous endoscopic gastrostomy tube was placed for the purposes of providing nutrition in the hopes of sustaining the patient to undergo further testing, and possibly cancer treatment if her neurologic status improved with plasma exchange. RS developed health-care–associated pneumonia as a result of her debility and extended hospital stay and was eventually transferred to a medical intensive care unit. At this point, according to her wishes, her husband requested that no further aggressive treatment be pursued. She was discharged home with hospice and expired approximately 1 week after discharge.

## IMPLICATIONS FOR THE ONCOLOGY ADVANCED PRACTITIONER

Paraneoplastic encephalopathy is a rare condition seen in patients with underlying cancer. Although immunomodulatory therapy such as corticosteroid administration and plasma exchange is sometimes helpful in reducing morbidity and mortality, most patients diagnosed with PND are unlikely to improve significantly due to deconditioning and debility at the time of diagnosis. The purpose of this case study is to raise awareness and understanding of this disease state. It is hoped that a broader understanding of paraneoplastic neurologic disorders will assist oncology APs in researching, educating our peers, and counseling our patients about signs and symptoms to monitor, so that we may promote earlier diagnosis and treatment of this devastating syndrome.
